# Complete genome sequence of *Pseudomonas aeruginosa* L3, a bacterium with Mn(II) oxidative property isolated from heavy metal-contaminated soils

**DOI:** 10.1128/mra.00026-24

**Published:** 2024-05-29

**Authors:** Lan Xue, Yueshuai Guo, Fengzhen Tang, Sha Chen, Fengfeng Liao, Ding Li

**Affiliations:** 1 School of Life Sciences and Chemistry, Hunan University of Technology, Zhuzhou, China; 2 Zhuzhou City Joint Laboratory of Environmental Microbiology and Plant Resources Utilization, Hunan University of Technology, Zhuzhou, China; 3 Hunan Provincial Engineering Research Center of Lily Germplasm Resource Innovation and Deep Processing, Hunan University of Technology, Zhuzhou, China; University of Southern California, Los Angeles, USA

**Keywords:** *Pseudomonas aeruginosa*, Mn(II) oxidation

## Abstract

*Pseudomonas aeruginosa* L3, isolated from heavy metal-contaminated soils, possesses the ability of Mn(II) oxidation. To further enhance the understanding of genes involved in Mn(II) oxidation, the complete genome of this strain was sequenced and annotated, which has a total size of 6.39 Mb with a G + C content of 66.39%.

## ANNOUNCEMENT

This strain L3 was originally isolated from heavy metal-contaminated soils near a smelter (113°09 '11.48 "E and 27°87' 07.41" N) in Zhuzhou, Hunan province, PR China, using the previously described methods by our group (1, 2). Upon comparison of the 16S rRNA sequences, it exhibits a complete similarity (100%) to members within *Pseudomonas aeruginosa* (3). Moreover, it possesses the ability to oxidize Mn(II) (3), which may have implications for the biogeochemical cycling of soil elements (4, 5). We hereby present the complete genome sequence of *P. aeruginosa* L3.

The strain L3 was cultured in a Luria-Bertani (LB) liquid medium at 35°C with a pH of 7.0 for 18 h. Genomic DNA was extracted using the Ezup Column Bacteria Genomic DNA Purification Kit (Sangon Biotech). The extracted DNA was used for library construction and subsequent sequencing on both an own-brand DNA nanoball sequencing (DNBSEQ) platform and a PacBio sequel II platform at the Beijing Genomics Institute (BGI, Shenzhen, China). On the DNBSEQ platform, a DNA sample (1 µg) was randomly fragmented using Covaris S220 (Covaris, USA) and size-selected to an average length of 200–400 bp using the Agencourt AMPure XP-Medium kit (Beckman Coulter). The MGIEasy Universal DNA Library Prep Set was employed to convert the fragmented DNA into a customized library for sequencing on the BGISEQ-500 system (6). We obtained 8,738,130 raw reads (the read length was 150 bp), with a total length of 1,310 million bp (Mb). After filtering low-quality reads using SOAPnuke v1.5.6 (7), 1,299 Mb of clean reads were obtained. On the PacBio platform, the genomic DNA was fragmentated using g-TUBE (Covaris), and the fragments ranging from 10 to 15 kb were selected. The SMRTbell express template prep kit 2.0 was used for library construction. Four arrays of zero-mode waveguides (ZMWs) of single molecule real-time sequencing were used to generate subreads, with subsequent removal of subreads shorter than 1,000 bp. PacBio sequencing produced 175,309 valid ZMWs and 1,436,085 subreads with a cumulative length of 16,562 Mb. The N_50_ and N_90_ lengths of these subreads were 12,434 and 8,131 bp, respectively. These subreads were assembled using the Canu program (8), while the clean reads from the DNBSEQ platform were employed for single-base corrections using the GATK program (9) during the assembly process. In addition, the overlaps were identified by the blastn algorithm and manually eliminated.

Collectively, the assembly yielded a single contig, which eventually gave rise to a total length of 6,389,938 bp of circular genome with a G + C content of 66.39%. The genome was rotated to *dna*A by Unicycler v0.4.7. The NCBI Prokaryotic Genome Annotation Pipeline (version 6.6 August 2023) was used for gene annotation. The genome was annotated by the methods of the best-placed reference protein set and GeneMarkS-2+ (v.1.14_1.25) (10), including 5,910 total genes and 5,787 coding genes. Structural and non-coding RNAs were annotated by Rfam (v.14.4) (11) and Infernal (v.1.1.1) (12), and tRNAs were annotated by tRNAscan-SE (v2.0.12) (13). Default parameters were used for all software unless otherwise noted. The principal genomic information can be found in Table 1.

**TABLE 1:** Genomic information for *P. aeruginosa* L3^*a*^

Bacterial strain	*P. aeruginosa* L3
SRA accession for DNBSEQ	SRX23848132
SRA accession for PacBio	SRX23256368
GenBank accession	CP142026
BGI DNBSEQ Data (Mb)	1,299 (a coverage of 203×)
PacBio Data (Mb)	16,562 (a coverage of 2,591×)
PacBio subreads mean length (bp)	11,533
PacBio subreads N50 (bp)	12,434
PacBio subreads N90 (bp)	8,131
Contig number	1
Genome size (bp)	6,389,938
GC content (%) of genome	66.39
GC content (%) of genes	67.14
Gene (total)	5,910
CDSs (total)	5,831
Genes (coding)	5,787
Genes (RNA)	79
rRNAs	4, 4, and 4 (5S, 16S, and 23S)
tRNAs	63
ncRNAs	4
CRISPR arrays	2

^*a*^ GC: Guanine and cytosine. CDS: Coding sequences. ncRNAs: Non-coding RNA.

## Data Availability

The complete genome sequence of *Pseudomonas aeruginosa* L3 has been deposited in NCBI GenBank under the BioProject accession number PRJNA1061838, the BioSample accession number SAMN39278653, and the GenBank accession number CP142026. The raw sequence data are available under Sequence Read Archive (SRA) accession numbers SRX23848132 (DNBSEQ) and SRX23256368 (PacBio), respectively.
